# A specific upregulated long noncoding RNA in colorectal cancer promotes cancer progression

**DOI:** 10.1172/jci.insight.158855

**Published:** 2022-08-08

**Authors:** Junshu Li, Yanhong Ji, Na Chen, Huiling Wang, Chao Fang, Xiaonan Yin, Zhiyuan Jiang, Zhexu Dong, Dan Zhu, Jiamei Fu, Wencheng Zhou, Ruiyi Jiang, Ling He, Zhang Hantao, Gang Shi, Lin Cheng, Xiaolan Su, Lei Dai, Hongxin Deng

**Affiliations:** 1 State Key Laboratory of Biotherapy and Cancer Center, West China Hospital, Sichuan University and Collaborative Innovation Center for Biotherapy, Chengdu, Sichuan, China.; 2 School of Pharmacy, Chengdu Medical College, Chengdu, Sichuan, China.; 3 Institute of Digestive Surgery, Sichuan University, and Department of Gastrointestinal Surgery, West China Hospital, West China School of Medicine, Sichuan University, Chengdu, China.; 4 Department of Biotherapy, Cancer Center, West China Hospital, Sichuan University, Chengdu, Sichuan, China.

**Keywords:** Cell Biology, Gastroenterology, Cell cycle, Colorectal cancer, Molecular biology

## Abstract

Long noncoding RNA (lncRNA) plays a crucial role in the pathogenesis of various diseases, including colorectal cancer (CRC). The gene mutations of adenomatous polyposis coli (APC) were found in most patients with CRC. They function as important inducers of tumorigenesis. Based on our microarray results, we identified a specific upregulated lncRNA in CRC (SURC). Further analysis showed that high SURC expression correlated with poorer disease-free survival and overall survival in patients with CRC. Furthermore, we found that mutated APC genes can promote the transcription of SURC by reducing the degradation of β-catenin protein in CRC. Functional assays revealed that knockdown of SURC impaired CRC cell proliferation, colony formation, cell cycle, and tumor growth. Additionally, SURC promotes CCND2 expression by inhibiting the expression of miR–185-5p in CRC cells. In conclusion, we demonstrate that SURC is a specific upregulated lncRNA in CRC and the SURC/miR–185-5p/CCND2 axis may be targetable for CRC diagnosis and therapy.

## Introduction

Colorectal cancer (CRC) is the third most common cancer in the world (1). It is estimated that there were more than 1.8 million new cases of CRC and 881,000 deaths from CRC in 2018 (2). The incidence of CRC is closely related to adenomatous polyposis coli (APC) gene mutation, which acts as a tumor suppressor gene (3). APC regulates the expression of Wnt target genes via promoting the degradation of β-catenin (4). The protein encoded by the WT APC gene promotes the degradation of β-catenin by binding with the β-catenin protein. Deletion or mutation of the APC gene leads to the accumulation of β-catenin in the nucleus, which activates the canonical Wnt signaling pathway (5). APC gene mutations were found in familial adenomatous polyposis (FAP) and 70%–80% of sporadic patients with CRC have APC gene inactivation (6). Thus, understanding the mechanism by which APC regulates the carcinogenesis and progression of CRC is necessary and of clinical significance.

Long noncoding RNA (lncRNA) is a class of noncoding RNA (ncRNA) with a length of more than 200 bp and without the ability to encode proteins (7). Recent studies have revealed that lncRNAs play a regulatory role in the progression of malignant cancers, such as breast cancer, gastric cancer, lung cancer, and CRC (8–11). In CRC, a series of lncRNAs was demonstrated as tumor suppressors and tumor promoters, which regulate gene expression and participate in the occurrence and development of CRC by regulating cell proliferation, cell cycle, epithelial-mesenchymal transition (EMT), drug resistance, and metastasis (12). LncRNA-SNHG11 promotes the invasion and metastasis of CRC cells by interacting with HIF-1α and activating the expression of HIF-1α (13). LncRNA-RPPH1 is upregulated in tissues of patients with CRC and induces CRC progression by inducing the EMT process (14). LINC01106 activates the expression of Gli family factors and drives the growth and stemness of CRC (15). Current research indicates that an APC-activated lncRNA-APC1 regulates the progression of CRC via noncanonical Wnt signaling (16). In recent years, a large number of lncRNAs have been identified using bioinformatics and high-throughput methods (17). However, there are still a large number of lncRNAs with unknown functions; thus, further research is required to determine new diagnostic and therapeutic targets.

Based on the previous microarray results of the azoxymethane/dextran sodium sulfate–induced (AOM/DSS–induced) colitis-associated cancer (CAC) model, we used weighted correlation network analysis (WGCNA) and lncRNA-mRNA coexpression network analysis to classify and predict the functions and signaling pathways of lncRNAs (18). We screened a lncRNA (lncRNA-AK028845) and found it was specifically upregulated in CRC; thus, we named it specific upregulated lncRNA in CRC (SURC). In the present study, we determined the expression and correlation with the prognosis of SURC in CRC and investigated the underlying mechanism that mediated the specific upregulation of SURC in CRC. The function of SURC in CRC was then explored, followed by the investigation of the molecular mechanism underlying the function. The findings provide a potentially novel perception for understanding the role of APC mutation in CRC progression and supply what we believe is a novel target for CRC diagnosis and prognosis prediction.

## Results

### Identifying a SURC.

**Figure 1:**
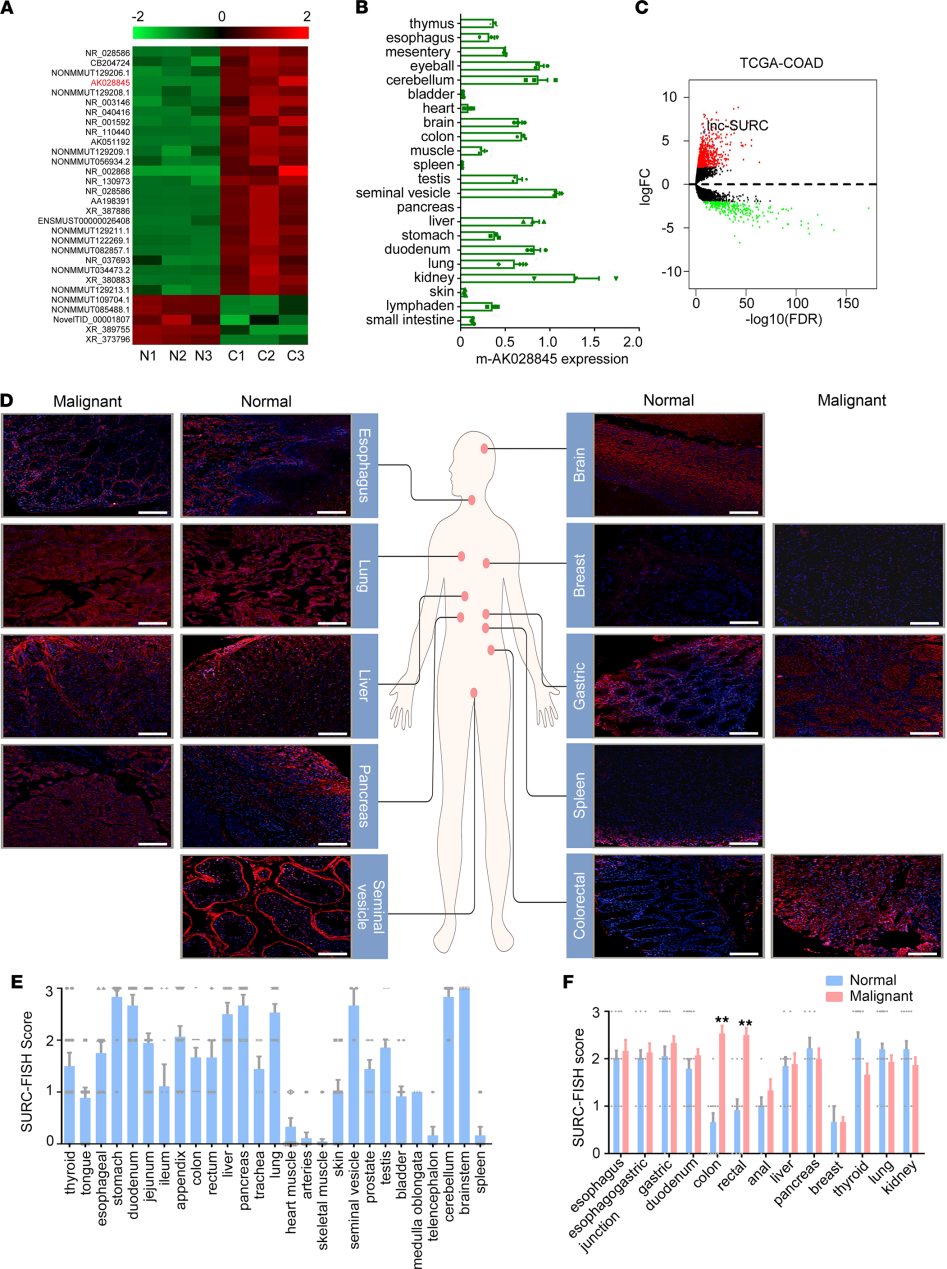
Identification of SURC as a specific upregulated lncRNA in CRC. (**A**) Heatmap for differentially expressed lncRNAs in AOM/DSS–induced colitis-associated cancer tissues compared with normal tissues in mice. (**B**) qPCR shows the expression of SURC in different tissues and organs of mice (*n* = 3). (**C**) The volcano plots show the fold changes and FDR values of 1174 lncRNA candidates in colon tumors (*n* = 469) versus adjacent normal tissues (*n* = 41) from the TCGA-COAD database. (**D**) FISH staining shows the distribution and expression of SURC in malignant and adjacent normal tissues among different organs. Scale bar: 100 μm. (**E**) SURC levels in various normal tissues by FISH assay. (**F**) SURC levels in malignant and adjacent normal tissues among the digestive system (**P < 0.01). Data are shown as the mean ± SEM. Statistical differences were calculated using an unpaired 2-tailed Student’s *t* test.

In a previous study we conducted, we determined the temporal expression profile of lncRNAs in CRC initiation (18). A comparison of lncRNA expression profile in colonic tissues between normal mice and the AOM/DSS–induced CAC model indicated that lncRNA-mAK028845 was significantly upregulated during CRC initiation (Figure 1A). Further results suggested that lncRNA-mAK028845 was widely expressed in normal tissue of mice (Figure 1B). Sequence homology alignment indicated that Homo sapiens lncRNA-AK028845 (named SURC) was located on chromosome 17 and contained the exon of the KRT37 gene and the pseudogene KRT41 (Supplemental Figure 1; supplemental material available online with this article; https://doi.org/10.1172/jci.insight.158855DS1). Furthermore, we screened the differentially expressed genes between colon cancer and adjacent tumors in The Cancer Genome Atlas (TCGA) database to clarify the similar expression profile of SURC between humans and mice. The volcano map indicated that SURC was significantly upregulated in malignant tissues of patients with CRC compared with adjacent normal colorectal tissues (Figure 1C). To determine the distribution and expression of SURC, FISH staining was employed to detect SURC in normal tissue microarrays and cancer tissue microarrays containing 13 kinds of solid tumors (Figure 1D). The results demonstrated that the expression of SURC was relatively high in the stomach, duodenum, liver, pancreas, lung, seminal vesicle, cerebellum, and brainstem. SURC was also moderately expressed in the thyroid, esophageal, jejunum, ileum, appendix, colon, rectum, trachea, prostate, and medulla oblongata, whereas in other tissues, such as the spleen, telencephalon, and muscle, the expression of SURC was low (Figure 1E). Further analysis demonstrated that SURC was highly expressed in a variety of tumors, including gastric cancer, lung cancer, and CRC (Figure 1F), but SURC was upregulated only in colon cancer tissues and rectal cancer tissues compared with adjacent normal tissues (Figure 1F). These results suggested that SURC was a specific upregulated lncRNA in CRC.

### SURC correlates with poor prognosis of patients with CRC.

**Figure 2:**
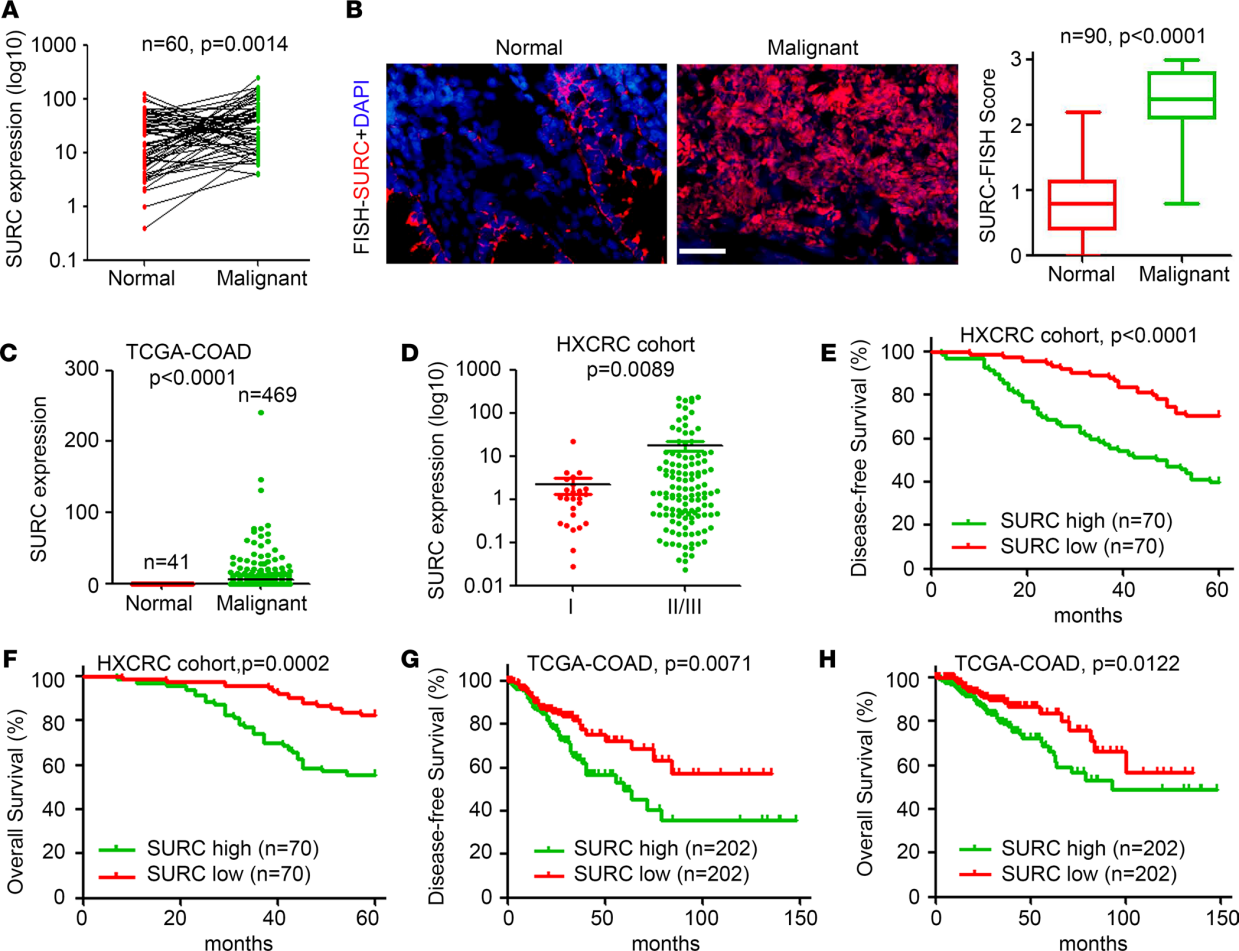
SURC upregulated in CRC and negatively correlated with prognosis. (**A**) Upregulation of SURC in malignant colorectal tissues compared with normal tissues by qPCR, *n* = 60. (**B**) Higher expression of SURC was detected in CRC samples than in matched normal tissues from HXCRC cohort (*n* = 90), which was measured by FISH. Analysis of FISH score of SURC in tissues. Scale bar: 100 μm. (**C**) Higher expression of SURC in CRC tissues (*n* = 469) than in adjacent normal tissues (*n* = 41) from the TCGA database. (**D**) Upregulation of SURC in stage II/III compared with stage I CRC tissues from HXCRC cohort. (**E**) Kaplan-Meier analysis of overall survival and (**F**) disease-free survival curves for CRC samples from our platform (*n* = 140) with SURC-low (*n* = 70) or SURC-high (*n* = 70) expression (Log-Rank test). (**G**) Kaplan-Meier plots of overall survival and (**H**) disease-free survival curves for patients with CRC from the TCGA database (*n* = 404), higher SURC expression with poorer prognosis. Data are shown as the mean ± SEM. Statistical differences were calculated using an unpaired 2-tailed Student’s *t* test for **A**–**D** and Log-Rank test (Kaplan-Meier curves) for **E**–**H**.

To investigate the expression and potential prognosis prediction role of SURC in CRC, quantitative PCR (qPCR) and FISH staining were employed to determine SURC expression in 150 surgical samples of patients with CRC. The qPCR results indicated that the expression of SURC in malignant tissues was significantly higher than that in adjacent normal colorectal tissues (Figure 2A). More SURC-positive cells were detected by FISH staining in CRC tissues compared with adjacent normal colorectal tissues (Figure 2B). The analysis of CRC samples in the TCGA database also confirmed the significant upregulation of SURC in malignant tissues of CRC compared with normal colorectal tissues (Figure 2C). qPCR analysis also suggested that SURC expression was significantly upregulated in most CRC cell lines such as SW480, HCT116, LoVo, and SW620 cells compared with normal intestinal epithelial cells (Supplemental Figure 2). Further analysis based on FISH staining in the HXCRC cohort (containing 140 pairs of CRC tissues) indicated that SURC was significantly more upregulated in patients in stage II/III than in patients in stage I (Figure 2D). In addition, we demonstrated that higher SURC expression was negatively correlated with poorer disease-free survival and overall survival in CRC (Figure 2, E and F). Similarly, the analysis in the TCGA Colon Cancer (TCGA-COAD) database also showed that patients with CRC with high SURC expression were associated with poorer disease-free survival and overall survival (Figure 2, G and H). Collectively, SURC was upregulated in CRC tissues and correlated with the poor prognosis of patients with CRC.

### APC mutation promoted SURC expression in CRC.

To investigate the molecular mechanism underlying specific upregulation of SURC in CRC, we first analyzed the expression correlation between SURC and KRT37, which contained a length of a similar sequence. The results indicated that there was a positive correlation between them in CRC samples (Supplemental Figure 3A). However, overexpression of KRT37 in LoVo and SW620 cells did not affect the expression of SURC (Supplemental Figure 3B). Meanwhile, knockdown of SURC did not significantly regulate the expression of KRT37 (Supplemental Figure 3C). These results indicated that there was no expression regulation between SURC and KRT37.

**Figure 3:**
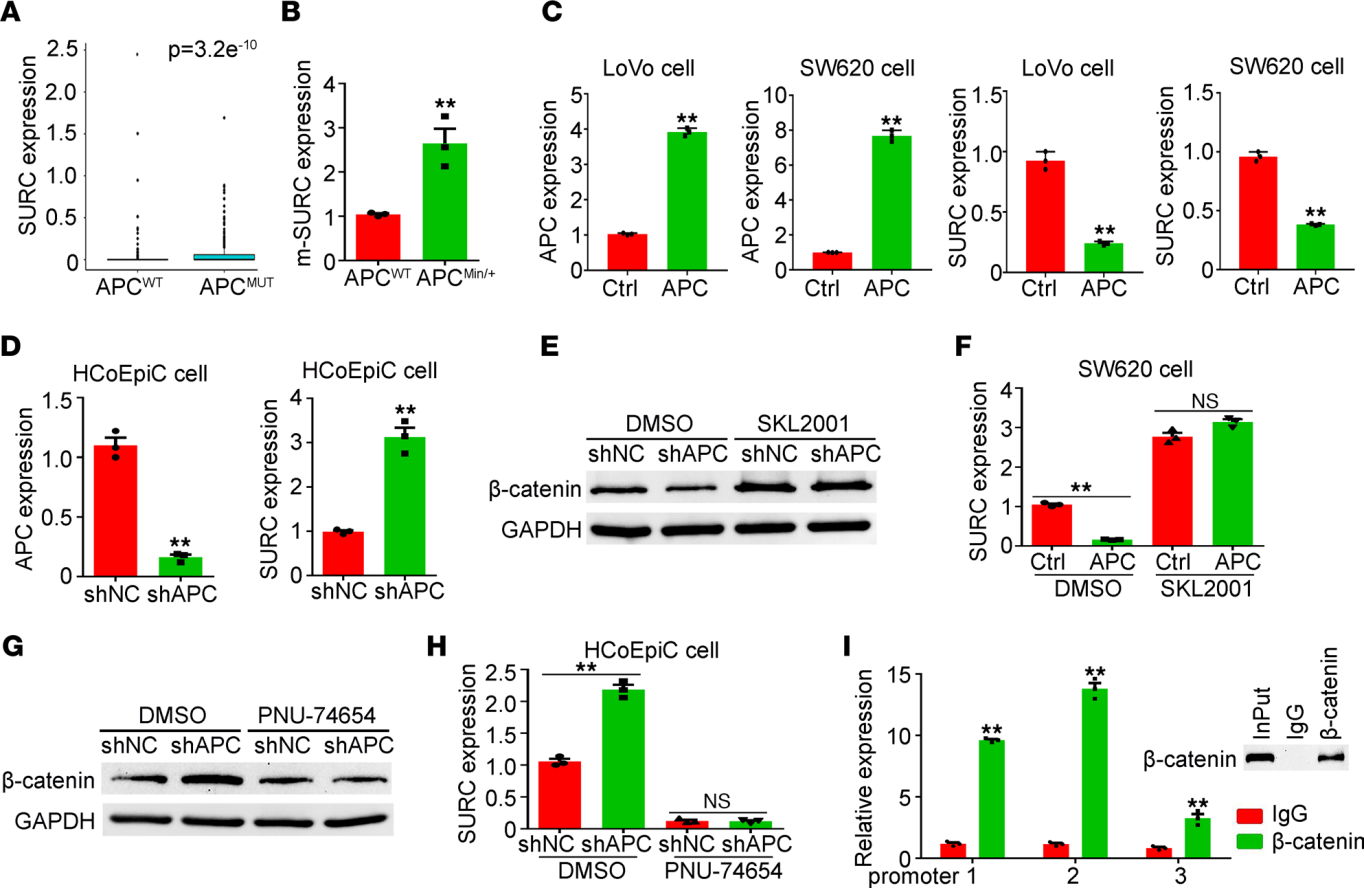
APC mutation promotes upregulation of SURC in CRC. (**A**) Analysis of SURC expression in the mutated APC tissues compared with WT tissues in TCGA database. (**B**) Detection of SURC expression in colorectal tissues of APC^WT^ and APC^Min/+^ mice (*n* = 3; **P < 0.01). (**C**) SURC expression was detected by qPCR in APC-overexpressing cells compared with control cells (*n* = 3; **P < 0.01). (**D**) SURC expression was detected by qPCR in HCoEpiC cells infected with lenti-shNC and lenti-shSURC (*n* = 3; **P < 0.01). (**E**) Western blotting shows the expression of β-catenin in SW620-Ctrl and SW620-APC cells treated with SKL2001 and DMSO. (**F**) qPCR shows the expression of SURC in SW620-Ctrl and SW620-APC cells treated with SKL2001 and DMSO (*n* = 3; **P < 0.01). (**G**) Western blotting shows the expression of β-catenin in HCoEpiC-shNC and HCoEpiC-shAPC cells treated with PNU-74654 and DMSO. (**H**) qPCR shows the expression of SURC in HCoEpiC-shNC and HCoEpiC-shAPC cells treated with PNU-74654 and DMSO (*n* = 3; **P < 0.01). (**I**) qPCR analyzes the expression of SURC promoter in products by ChIP assay in LoVo cells (*n* = 3; **P < 0.01). Data are shown as the mean ± SEM. Single comparisons to Ctrl were made using unpaired 2-tailed Student’s *t* test for **A**–**D**, **F**, **H**, and **I**. Ctrl, Control.

Next, we analyzed the correlation between SURC expression and the most frequently mutated genes in CRC based on the TCGA-COAD database and demonstrated that the expression of SURC was significantly upregulated in APC-mutated CRC samples compared with APC WT samples (Figure 3A and Supplemental Figure 3D). Higher expression of murine SURC was also detected in colorectal tissues of APC^Min/+^ mice compared with APC^WT^ mice (Figure 3B). Ectopic expression of WT APC in SW620 and LoVo cells significantly inhibits SURC expression (Figure 3C). Meanwhile, the knockdown of APC in human colon epithelial cells dramatically promoted SURC expression (Figure 3D). APC is a negative regulatory factor of β-catenin, which functions as a transcription factor. Thus, we speculated that APC mutation led to the increased expression of SURC via promoting β-catenin expression in CRC. To clarify if β-catenin had regulatory effects on SURC expression, we treated CRC cells with PNU-74654 (an inhibitor of β-catenin) and SKL2001 (an activator of β-catenin), respectively. We found that the decreased expression of β-catenin inhibited the expression of SURC, while the activated β-catenin protein level promoted SURC expression (Supplemental Figure 3, E and F). Furthermore, activation of β-catenin efficiently attenuated APC-mediated downregulation of SURC in SW620 cells (Figure 3, E and F), while the inhibition of β-catenin efficiently blocked APC knockdown-induced SURC upregulation in human colon epithelial cells (Figure 3, G and H). Further informatics analysis based on the PROMO database (http://alggen.lsi.upc.es/cgi-bin/promo_v3/promo/promoinit.cgi?dirDB=TF_8.3) predicted that β-catenin could bind to SURC promoter sites, and ChIP results in LoVo cells confirmed the binding efficiency of β-catenin on the promoters of SURC (Figure 3I). These results indicated that APC mutation increased the expression of SURC by promoting the binding of β-catenin with SURC promoter.

### Knockdown of SURC inhibits CRC growth.

**Figure 4:**
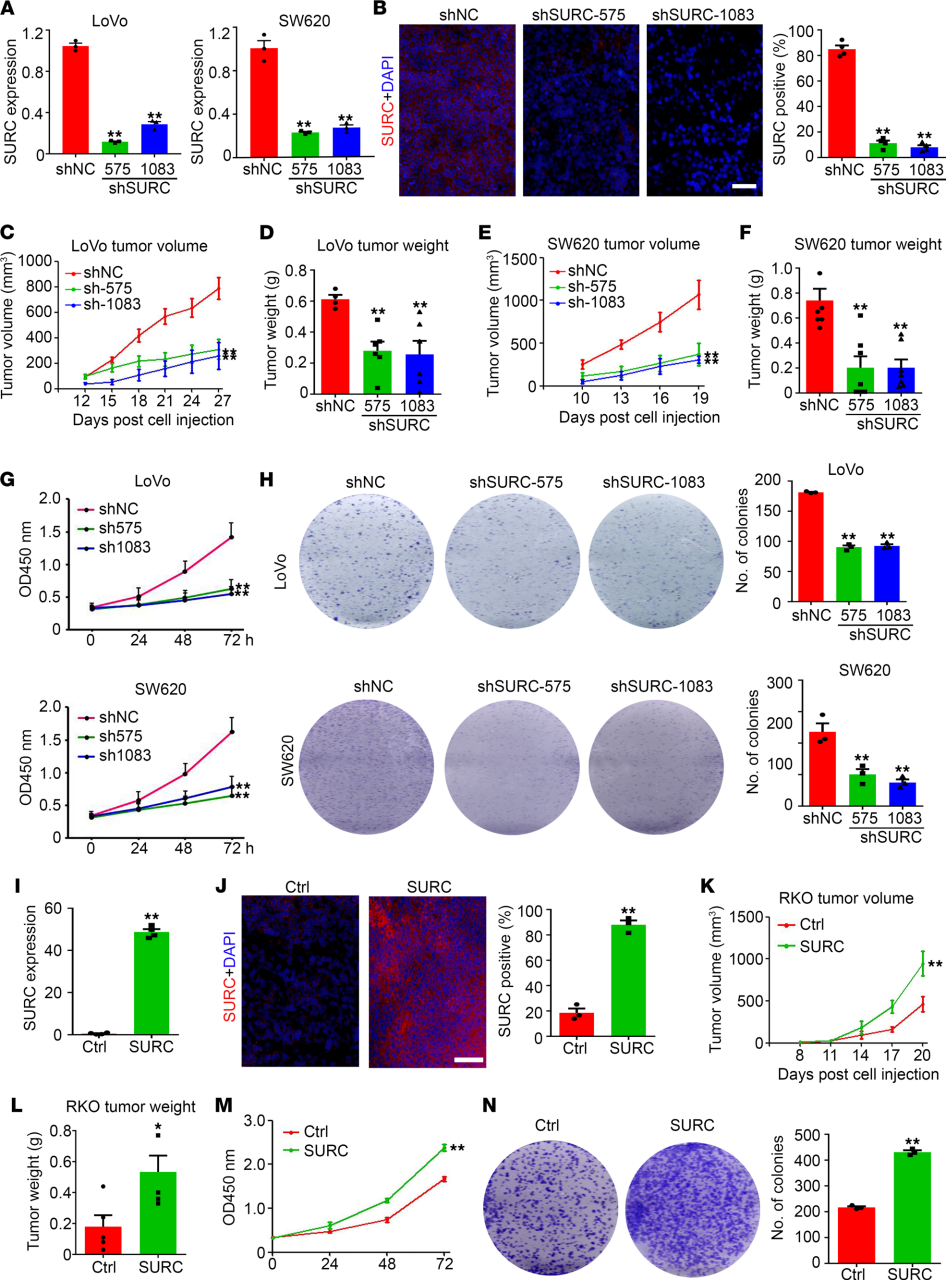
SURC promotes cell proliferation and tumor growth in vivo and in vitro. (**A**) SW620 and LoVo cells were transfected with shNC or shSURC and detected by qPCR (*n* = 3; **P < 0.01). (**B**) Detection of SURC expression in s.c. tumors by FISH. Scale bar: 100 μm. Analysis of SURC positive cells in each frame (*n* = 3; **P < 0.01). (**C**–**F**) Tumor volume and tumor weight were measured in SW620 and LoVo tumors and compared among groups (*n* = 6; **P < 0.01). (**G**) CCK8 assay shows relative cell growth at 0, 24, 48, and 72 hours (*n* = 5; **P < 0.01). (**H**) The colony formation pictures of SW620 and LoVo cells infected with shNC and shSURC. A total of 3 biologically independent experiments were performed (*n* = 3; **P < 0.01). (**I**) RKO cells were infected with lenti-SURC and detected by qPCR (*n* = 3; **P < 0.01). (**J**) FISH staining shows SURC expression of xenograft tumors from mice injected s.c. with control or SURC-overexpressing RKO cells. Scale bar: 100 μm. Analysis of SURC positive cells in each frame (*n* = 3; **P < 0.01). (**K** and **L**) Tumor volume and tumor weight were measured in RKO tumors among various groups (*n* = 5; *P < 0.05, **P < 0.01). (**M**) CCK8 assay shows relative cell proliferation at 0, 24, 48, and 72 hours in RKO cells (*n* = 5; **P < 0.01). (**N**) Effects of SURC overexpression on colony formation in RKO cells (*n* = 3; **P < 0.01). Data are shown as the mean ± SEM. Statistical differences were calculated using 1-way ANOVA and Dunnett’s multiple-comparison test for **A**–**H** and unpaired 2-tailed Student’s *t* test for **I**–**N**. Ctrl, Control.

To clarify the regulatory effect of SURC on the growth of CRC, we stably knocked down SURC in SW620 and LoVo cells through lentivirus-mediated shRNA targeting SURC (Figure 4A). The cells were then injected into mice for s.c. tumor model establishment, and the results confirmed the decrease of SURC in SURC knockdown tumor tissues (Figure 4B, and Supplemental Figure 4, A and B). Our results suggested that knockdown of SURC significantly inhibits LoVo tumor growth by 60.7% (shSURC-575) and 67.3% (shSURC-1083) in tumor volume (Figure 4C) and by 53.8% (shSURC-575) and 66.7% (shSURC-1083) in tumor weight (Figure 4D). Similar inhibition of tumor volume and tumor weight were also observed in SW620 tumors (Figure 4, E and F). In vitro results indicated that silencing SURC expression effectively inhibits CRC cell proliferation (Figure 4G) and colony formation (Figure 4H). After that, we obtained RKO cells that stably overexpressed SURC to further determine the function of SURC. Then, we detected upregulation of SURC in RKO cells (Figure 4I) and s.c. tumors (Figure 4J, and Supplemental Figure 4C) by qPCR and FISH staining. Consistent with the previous results, we found that overexpression of SURC significantly promotes the growth of s.c. tumors (Figure 4, K and L) and increases cell proliferation (Figure 4M) and colony formation of RKO cells (Figure 4N). These results indicated that SURC functions as an oncogene in CRC.

### SURC directly binds miR–185-5p.

**Figure 5:**
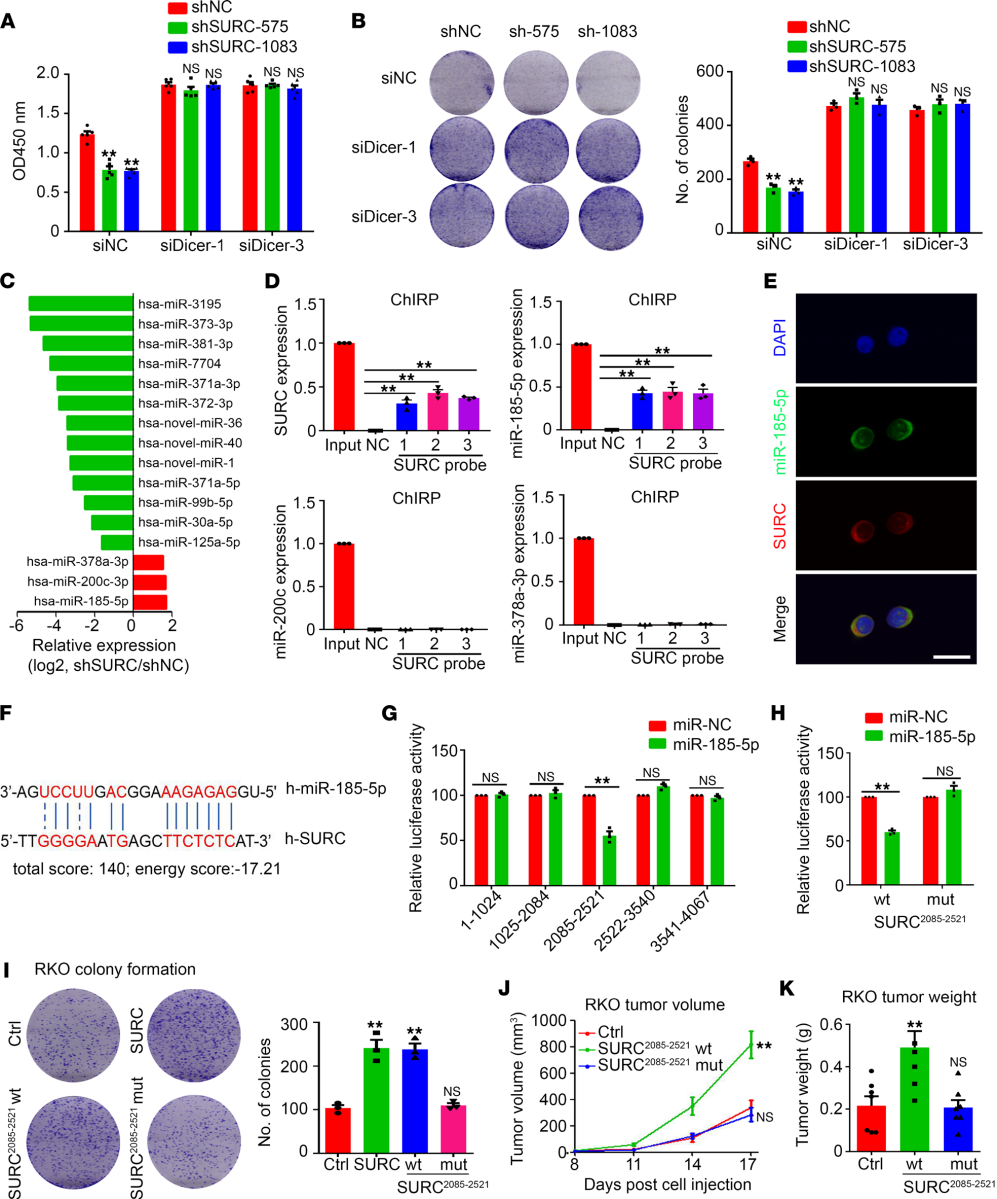
Direct binding of SURC to miR–185-5p. (**A**) CCK8 assay shows the relative cell growth in SW620 cells transfected with siNC or siDicer (*n* = 5; **P < 0.01). (**B**) Colony formation of SW620 cells transfected with siNC or siDicer (*n* = 3; **P < 0.01). (**C**) Altered microRNA expression in shSURC or shNC-treated LoVo cells by RNA-Seq. (**D**) ChIRP assay detected the ability of SURC direct binding to miRNA in SW620 cells (*n* = 3; **P < 0.01). (**E**) FISH assay shows the colocalization of SURC and miR–185-5p. Scale bar: 10 μm. (**F**) Prediction of binding sites of SURC and miR–185-5p by bioinformatics analysis. (**G**) The dual-luciferase reporter system confirmed 2085-2521 segment of SURC was combined with miR–185-5p (*n* = 3; **P < 0.01). (**H**) The dual-luciferase reporter system shows the combination between miR–185-5p and SURC core region transfected with SURC^2085-2521^WT or MUT plasmid (*n* = 3; **P < 0.01). (**I**) Colony formation shows the function of SURC core region in RKO cells which was transfected with SURC^2085-2521^WT or MUT plasmid (*n* = 3; **P < 0.01). (**J**) Tumor volume and (**K**) tumor weight were measured in RKO cells infected with SURC^2085-2521^ MUT compared with WT cells (*n* = 7; **P < 0.01). Data are shown as the mean ± SEM. Differentially expressed miRNAs were defined when the fold change was greater than or equal to 1 and adjusted *P* value less than 0.05 between different groups. Statistical differences were calculated using 1-way ANOVA and Dunnett’s multiple-comparison test (for **A**, **B**, **D**, and **I**–**K**) and unpaired 2-tailed Student’s *t* test (for **G** and **H**).

To determine whether SURC promotion of the growth of CRC depends on the presence of miRNA, we transfected SW620 and LoVo cells with siRNA targeting Dicer (Supplemental Figure 5A). Cell proliferation assay and colony formation assay indicated that blocking miRNA synthesis by knockdown of Dicer efficiently attenuates the regulation of SURC on the growth and colony formation of CRC cells (Figure 5, A and B). These results suggested that SURC promoted the growth of CRC depending on miRNA.

To find the target miRNA interacting with SURC, we detected the miRNA expression profiles in shSURC and shNC-treated LoVo cells. The results suggested that 13 miRNAs were downregulated and 3 miRNAs (miR–185-5p, miR-220c, and miR–378a-3p) were upregulated in shSURC LoVo cells compared with control (Figure 5C). Furthermore, ChIRP experiments showed that SURC directly targeted miR–185-5p rather than miR-220c and miR–378a-3p (Figure 5D). FISH staining results indicated that SURC was mainly located in the cytoplasm of SW620 and LoVo cells (Supplemental Figure 5B), which was further confirmed by qPCR results based on nuclear cytoplasmic separation (Supplemental Figure 5C). As shown in Figure 5E, we demonstrated that SURC and miR–185-5p colocalized in the cytoplasmic of CRC cells by FISH staining (Figure 5E). Target prediction suggested that miR–185-5p directly binds the 2311-2333 region of SURC, which contains 14 binding sites (Figure 5F). Dual-luciferase reporter results show that miR–185-5p inhibits the transcription of SURC (Supplemental Figure 5D). Then, SURC was divided into 5 truncated vectors based on the predicted binding sites of miR–185-5p, and the results suggested that SURC (2085-2521 region) was the binding region of miR–185-5p by dual-luciferase reporter assay (Figure 5G). The transcription of SURC with mutated binding sites of miR–185-5p was not affected by miR–185-5p in CRC cells (Figure 5H). We next constructed lentivirus-mediated WT and mutated overexpression plasmids of the 2085-2521 region that were used to infect RKO cells. The results showed that overexpression of WT SURC^2085-2521^ significantly promotes cell proliferation, colony formation, and s.c. tumor growth, while overexpression of the mutant SURC^2085-2521^ rescues this increase (Figure 5, I–K, and Supplemental Figure 5E). These results suggested that miR–185-5p was a direct target for SURC to promote the growth of CRC.

### SURC inhibits miR–185-5p expression in CRC.

**Figure 6:**
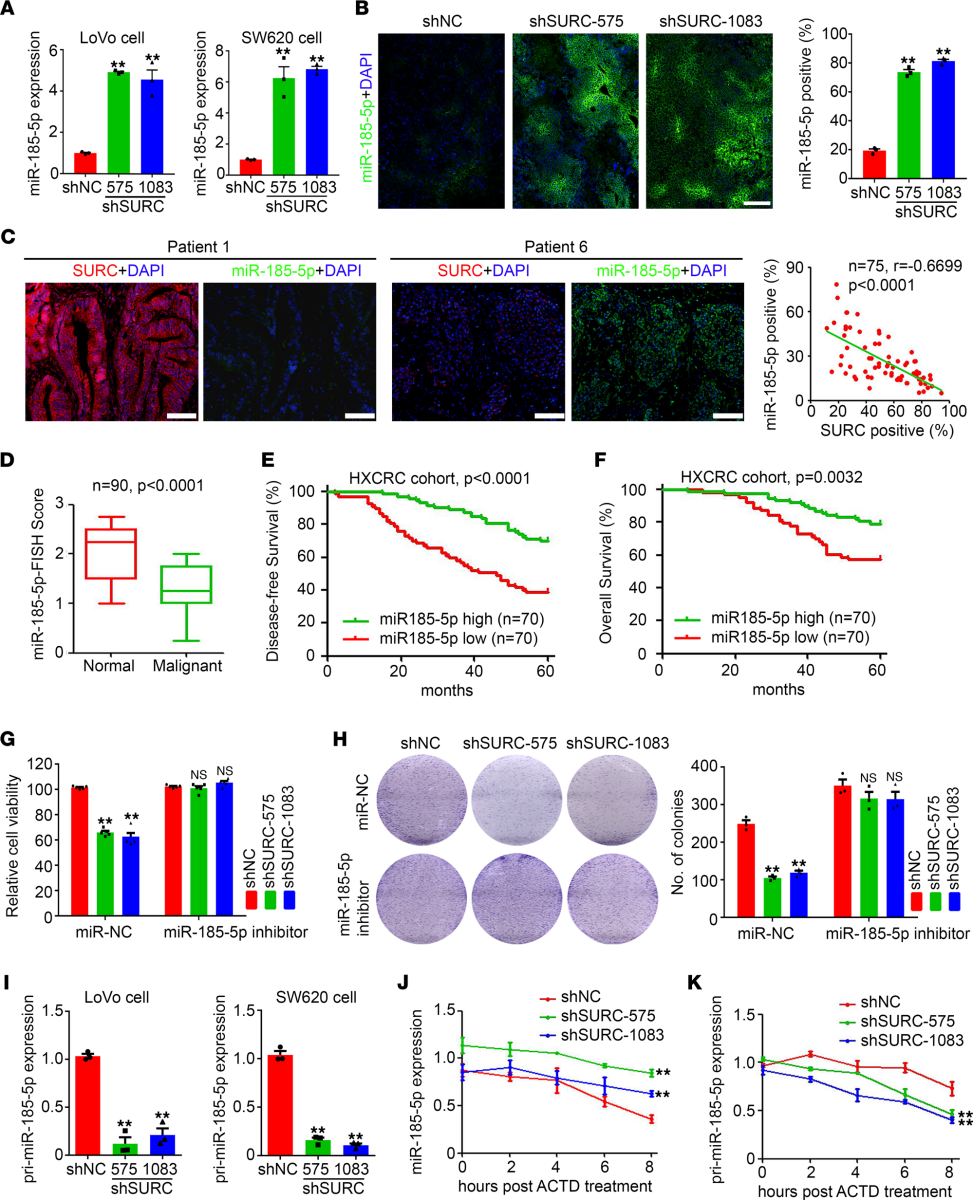
SURC modulated CRC progression by regulating the expression of miR–185-5p. (**A**) qPCR shows the expression of miR–185-5p in LoVo and SW620 cells (*n* = 3; **P < 0.01). (**B**) The FISH assay shows the expression of miR–185-5p in s.c. tumor tissue of SW620 cells. Scale bar: 100 μm. Analysis of miR–185-5p positive cells in each frame (*n* = 3; **P < 0.01). (**C**) Correlations between the SURC levels and the miR–185-5p levels in patients with CRC of HXCRC cohort (*n* = 75) were determined by FISH staining. Scale bar: 200 μm. The *r* values and *P* values are from Pearson’s correlation analysis. (**D**) Lower expression of miR–185-5p was detected in CRC samples than in matched normal tissues from HXCRC cohort (*n* = 90), which was measured by FISH. (**E**) Kaplan-Meier plots of overall survival and (**F**) disease-free survival for CRC samples from HXCRC cohort (*n* = 140). (**G**) Cell viability (*n* = 5; **P < 0.01) and (**H**) colony formation (*n* = 3; **P < 0.01) were examined after administration with miR–185-5p inhibitor. (**I**) qPCR shows the expression of pri-miR–185-5p in LoVo and SW620 cells infected with lenti-shNC and lenti-shSURC (*n* = 3; **P < 0.01). (**J**) Levels of miR–185-5p and (**K**) pri-miR–185-5p were examined at different times after administration with actinomycin d in SW620 cells (*n* = 3; **P < 0.01). Data are shown as the mean ± SEM. Statistical differences were calculated using 1-way ANOVA and Dunnett’s multiple-comparison test for **A**, **B**, and **G**–**K**, unpaired 2-tailed Student’s *t* test for **D**, and Log-Rank test (Kaplan-Meier curves) for **E** and **F**.

Based on the above findings, we intended to further clarify the regulatory effect of SURC on miR–185-5p expression. We demonstrated that knockdown of SURC significantly upregulated miR–185-5p expression in CRC cells and s.c. tumors (Figure 6, A and B, and Supplemental Figure 6A), while ectopic expression of SURC dramatically inhibited miR–185-5p expression in RKO cells and s.c. tumors (Supplemental Figure 6, B and C). miR–185-5p expression was also determined in 75 malignant tissues of patients with CRC and low miR–185-5p expression was found in SURC high expression tissues (Figure 6C). Further analysis demonstrated the negative correlation between SURC expression and miR–185-5p expression in malignant tissues of patients with CRC (Figure 6C). Based on the miR–185-5p determination in CRC tissues, we demonstrated that miR–185-5p expression was significantly decreased in CRC tissues compared with normal tissues (Figure 6D) and miR–185-5p expression was positively correlated with disease-free survival and overall survival of patients with CRC (Figure 6, E and F). To clarify the potential necessary role of miR–185-5p underlying SURC regulation of the growth of CRC, we treated the cells with miR–185-5p inhibitor (Supplemental Figure 6D) and demonstrated that miR–185-5p inhibitor efficiently rescues the regulation role of SURC on CRC cell proliferation and colony formation (Figure 6, G and H, and Supplemental Figure 6E). These results indicated that miR–185-5p plays a necessary role during SURC in promoting CRC growth.

To further investigate the mechanism under SURC inhibiting miR–185-5p expression, we detected the expression of primary transcripts of microRNA 185-5p (pri-miR–185-5p). Our results suggested that the expression of pri-miR–185-5p was significantly decreased in SURC knockdown CRC cells (Figure 6I). Further ChIRP assay showed that SURC was also directly targeted with pri-miR–185-5p (Supplemental Figure 6F). The effect of SURC on miR–185-5p degradation was then investigated by adding the RNA synthesis inhibitor actinomycin d. Our results suggested that knockdown of SURC inhibited the degradation of miR–185-5p in CRC cells (Figure 6J), while knockdown of SURC promoted the synthesis of miR–185-5p from pri-miR–185-5p (Figure 6K). Moreover, we found that overexpression of SURC^2085-2521^ WT plasmid promoted the degradation of miR–185-5p in SURC knockdown SW620 cells compared with SURC^2085-2521^ mutant (MUT) plasmid, and overexpression of SURC^2085-2521^ WT plasmid inhibited the synthesis of miR–185-5p from pri-miR–185-5p (Supplemental Figure 6, G and H). Collectively, knockdown of SURC promotes miR–185-5p expression by inhibiting mature miR–185-5p degradation and promoting the synthesis of mature miR–185-5p from pri-miR–185-5p in CRC cells.

### SURC regulates the activity of the miR–185-5p/CCND2 axis.

**Figure 7:**
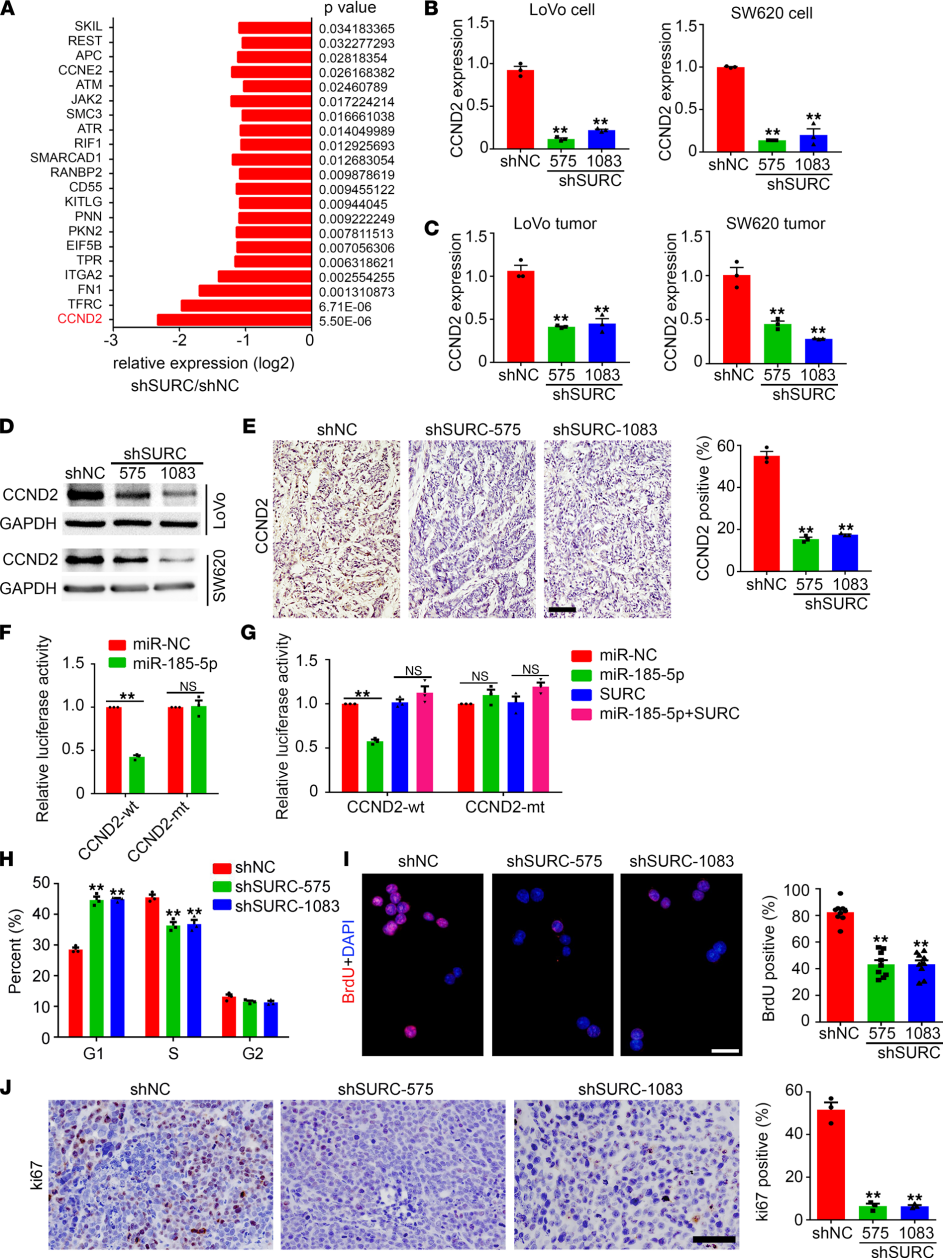
SURC regulated CRC cell cycle by affecting CCND2 expression. (**A**) SURC-regulated gene expression by RNA-Seq. (**B**) qPCR shows the expression of CCND2 in LoVo and SW620 cells infected with lenti-shNC and lenti-shSURC (*n* = 3; **P < 0.01). (**C**) qPCR shows the expression of CCND2 in s.c. tumors of LoVo and SW620 cells (*n* = 3; **P < 0.01). (**D**) Western blotting shows the expression of CCND2 in LoVo and SW620 cells. GAPDH was used as a loading control. (**E**) IHC staining of CCND2 in the s.c. tumors of shNC and shSURC cells. Analysis of CCND2-positive cells (*n* = 3; **P < 0.01). (**F**) Dual-luciferase reporter assays to test the interaction between miR–185-5p and CCND2 by using a synthetic miR–185-5p mimic cotransfected with CCND2-wt or CCND2-mt constructs (*n* = 3; **P < 0.01). (**G**) Dual luciferase assay of SW620 cells cotransfected with the CCND2 reporter constructs (wt or mt), the SURC overexpressing plasmids and miR–185-5p mimic (*n* = 3; **P < 0.01). (**H**) The cell cycle was analyzed by flow cytometry analysis in SW620 cells (*n* = 3; **P < 0.01). (**I**) BrdU assays of the SW620 cells with SURC knockdown by shRNAs compared with the control. (Scale bar: 100 μm; *n* = 3; **P < 0.01). (**J**) IHC staining of Ki67 in the shNC and shSURC s.c. tumors. Scale bar: 100 μm. Analysis of Ki67-positive cells in each frame (*n* = 3; **P < 0.01). Data are shown as mean ± SEM. Differentially expressed mRNAs were defined when the fold change was greater than or equal to 1 and adjusted *P* value was less than 0.05 between different groups. Statistical differences were calculated using 1-way ANOVA and Dunnett’s multiple-comparison test (for **B**, **C**, **E**, and **H**–**J**) and unpaired 2-tailed Student’s *t* test (for **F** and **G**). mt, mutant.

To further elucidate the downstream targets of SURC in regulating CRC growth by targeting miR–185-5p, we detected the mRNA expression profiles in SURC knockdown and control cells and found that SURC knockdown decreases the expression of a series of coding genes that were involved in the p53 signaling pathway, PI3K-Akt signaling pathway (Supplemental Figure 7, A and B). CCND2 is 1 of the significantly downregulated genes (Figure 7A), and the decrease of CCND2 was confirmed by qPCR in SURC knockdown CRC cells (Figure 7B) and s.c. tumor tissues (Figure 7C). Western blotting results suggested that knockdown of SURC inhibits CCND2 expression in CRC cells (Figure 7D). Additionally, fewer CCND2 positive cells were observed in SW620-shSURC tumors (Figure 7E). Luciferase report assay indicated that miR–185-5p inhibits the transcription of CCND2 (Figure 7F), while SURC efficiently blocks the inhibitory effect of miR–185-5p on CCND2 transcription (Figure 7G). The cell cycle and proliferative ability of CRC cells were then investigated. Cell cycle analysis indicated that knockdown of SURC prevents CRC cells from transforming from the G1 phase to the S phase (Figure 7H, and Supplemental Figure 7C). BrdU staining indicated that knockdown of SURC inhibits the proliferative activity of CRC cells (Figure 7I). Fewer ki67 positive cells were also detected in SW620-shSURC tumors (Figure 7J). These studies indicated that SURC promotes CRC growth and cell proliferation via regulating the activity of miR–185-5p/CCND2.

## Discussion

**Figure 8:**
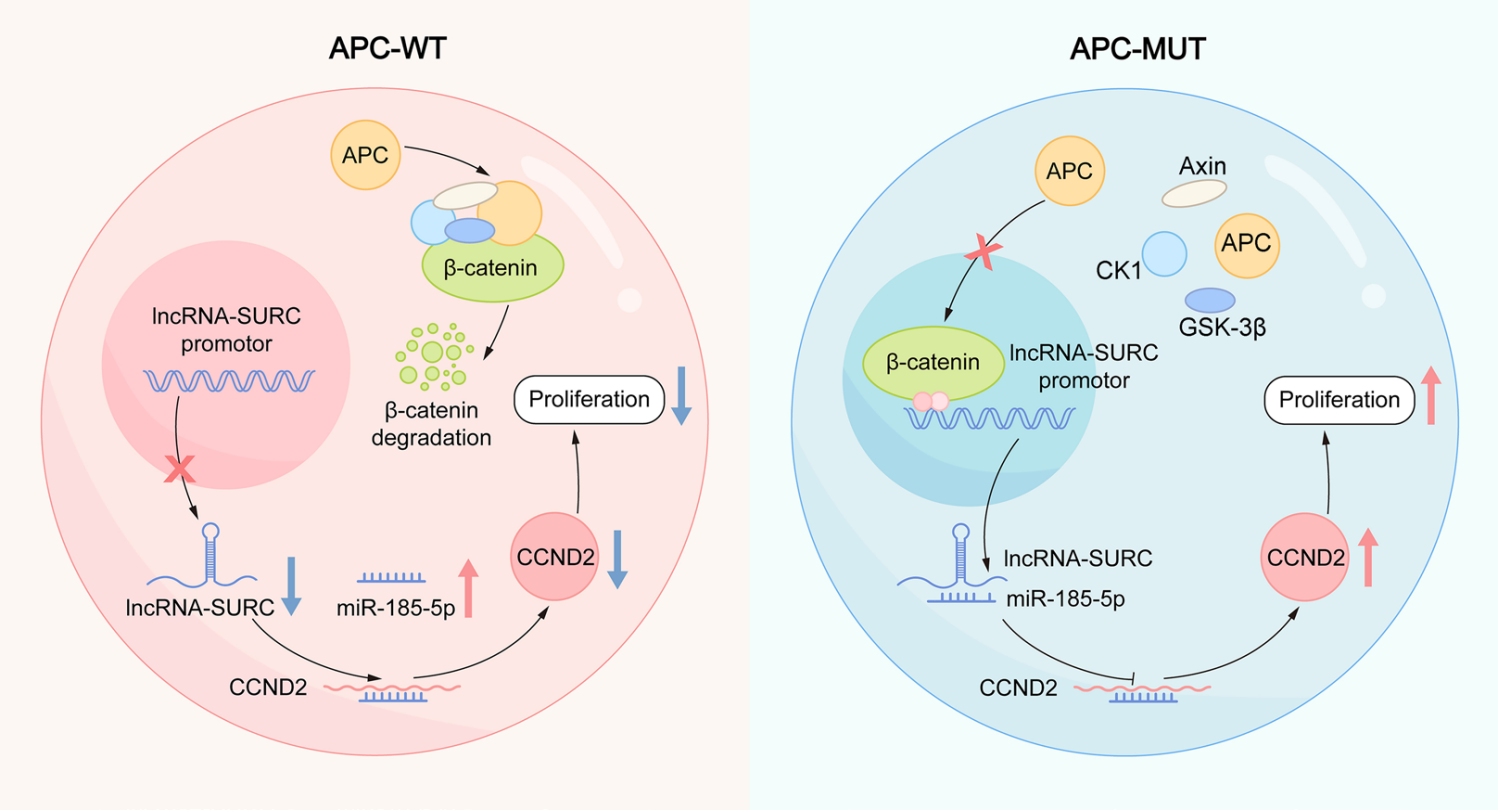
Molecular mechanism model for SURC in CRC cells. Mutated APC protein resulted in stabilization of β-catenin in CRC, which promotes the transcription of SURC via binding to its promoter (right), while WT APC protein caused the degradation of β-catenin, which inhibits the transcription of SURC (left). After transcription, SURC was transferred to the cytoplasm and inhibits miR–185-5p expression via binding to miR–185-5p, which results in CCND2 expression, cell proliferation, and tumor growth.

In the present study, we identified what we believe is a novel lncRNA (SURC) that is specifically upregulated in CRC and functions as an oncogene. A mutated APC protein resulted in stabilization of β-catenin in CRC, which promotes the transcription of SURC via binding to its promoter (Figure 8), while the WT APC protein caused the degradation of β-catenin, which inhibits the transcription of SURC (Figure 8). After transcription, SURC was transferred to the cytoplasm and inhibits miR–185-5p expression via binding to miR–185-5p, which results in CCND2 expression, cell proliferation, and tumor growth (Figure 8). High expression of SURC was demonstrated in malignant tissues of patients with CRC, and SURC expression was correlated with poorer disease-free survival and overall survival.

Previous studies have found that many lncRNAs such as CCAT2 (19, 20) and LncGata6 (21) are significantly upregulated in CRC compared with normal colorectal tissues. In the present study, we demonstrated that SURC was widely expressed in various solid tumor tissues. However, further analysis suggested that SURC was only significantly upregulated in CRC but not in other solid tumor tissues when compared with paired adjacent normal tissues. These findings indicated that SURC expression would be an efficient diagnosis target for CRC. However, further investigations based on large samples should be performed to determine the possibility, especially for the SURC expression in blood. Meanwhile, our study also indicated that high expression of SURC in tumor tissues was correlated with poorer disease-free survival and overall survival, which suggested that SURC is a predictor for prognosis of patients with CRC.

The APC gene is a tumor-suppressor gene and plays an important role in cell proliferation, migration, adhesion, differentiation, and chromosome aggregation in CRC (22–24). The study by Wang et al. showed that WT APC-activated lncRNA (lncRNA-APC1) was downregulated in CRC compared with normal colorectal tissues and inhibited cell growth, angiogenesis, and metastasis (16). However, APC mutation, but not APC downexpression, was found in most sporadic CRCs (25, 26), and APC mutation was one of the most important inducers for CRC tumorigenesis and progression (27). In the present study, we demonstrated that mutated APC protein mediated stabilization of β-catenin protein and promoted the transcription of SURC via binding to its promoter. Thus, due to the high mutation frequency (78%–80%) of APC in CRC and the low mutation frequency of APC in other solid cancers (28–31), specific upregulation of SURC was demonstrated only in CRC but not in other solid tumor tissues. The high expression of SURC in other solid tumor tissues and paired normal tissues may contribute to the base expression of the β-catenin protein.

In recent years, an increasing number of studies have shown that lncRNAs played a key role in regulating the biological process of CRC. The altered expression of lncRNA-NEAT1 led to changes in cell proliferation, invasion, and migration in both in vivo and in vitro experiments (32). LncRNA-SNHG15 regulated the expression of downstream genes including MYC, NRAS, BAG3, and ERBB3, which are closely related to cancer progression (33, 34). As a new definition of lncRNA, the SURC function was unclear. In the present study, we demonstrated that knockdown of SURC impaired CRC tumor growth in mice and CRC cell proliferation in vitro. The ectopic expression of SURC promoted the growth of s.c. transplanted tumors in mice. These findings clarified the function of SURC and provided a potential therapy target for CRC.

LncRNAs regulated the tumorigenesis and progression of the tumor through a variety of mechanisms, including acting as microRNA sponges, modulating post-transcription levels, and regulating the expression of proximal genes and distal genes in the nucleus (35–37). In the present study, based on miRNA expression sequence, ChIRP, and FISH staining, we demonstrated that SURC directly interacts with miR–185-5p, which functioned as a tumor suppressor in various cancers (38–40). We also demonstrated that miR–185-5p expression was downregulated in SURC–expressed CRC cells, which may contribute to the degradation of mature miR–185-5p via directly targeting with miR–185-5p and the inhibition of synthesis of miR–185-5p from pri-miR–185-5p via the direct interaction between SURC and pri-miR–185-5p. The molecular mechanism underlying the process is confusing and interesting, and further investigations are needed to demonstrate it. Further experiments with miR–185-5p inhibitor also confirmed the necessary role of miR–185-5p during SURC in promoting CRC growth. Several coding genes were demonstrated as the direct targets of miR–185-5p (41–43), and the results of sequencing and deregulated gene analysis indicated that CCND2 was inhibited in SURC knockdown CRC cells. Ectopic expression of SURC efficiently blocked miR–185-5p–mediated inhibition of CCND2 transcription, which suggested that SURC promotes CRC growth and cell proliferation via regulating the activity of miR–185-5p/CCND2. These potentially novel findings provide solid evidence for understanding the mechanism underlying SURC regulation of CRC growth.

## Methods

### Clinical specimens.

The experiments were approved by the West China Hospital of Sichuan University Biomedical Research Ethics Committee (year 2018 [serial number 280]). Clinical samples were collected between 2013 and 2014 at the Department of Gastrointestinal Surgery, West China Hospital, Sichuan University. All patients were histologically diagnosed with cancer, underwent surgery, and followed up regularly after the operation. All the frozen tissues were fixed with 4% paraformaldehyde, embedded with paraffin, and cut into 4 μm slides for H&E staining. The histopathological evaluation was performed by an experienced pathologist and reviewed by 2 other pathologists. The qualified samples were used for tissue microarray by Outdo Biotech and the tissue microarray was named the HXCRC cohort. The disease-free survival and overall survival were recorded by arranging follow-ups during the 5 years after surgery.

### Animal experiment.

In vivo experiments were performed in compliance with Sichuan University guidelines concerning animal use and care. Female BALB/c nude mice (4–5 weeks) were purchased from Beijing HFK Bioscience. All mice were grown in a pathogen-free condition with free access to water and food. For the tumor xenograft experiments, 5 × 10^6^ SW620, LoVo, and RKO cells were injected s.c. into the ventral s.c. mice. At 10 days after cell injection, the tumor length and width were measured by a vernier caliper every 3 days. The tumor volume was calculated as tumor volume = length × width^2^ × 0.52. After the s.c. tumor grew well, the mice were euthanized, and the tumors were dissected and weighed.

### Cell culture and treatment.

SW620, 293T, HCoEpiC, and RKO cells were cultured in DMEM containing 10% FBS (MilliporeSigma) and 1% penicillin/streptomycin. F-12K Nutrient Mixture (Invitrogen) was used to cultivate LoVo cells with 10% FBS and 1% penicillin/streptomycin. All cells were purchased from American Type Culture Collection and were detected by the mycoplasma detecting kit (Yise Medical Technology) to confirm a nonmycoplasma condition. All cells were cultured in the condition of 37°C and 5% CO_2_. The small interfering RNAs used in this study were siDicer-1, siDicer-2, and siDicer-3, which were purchased from RiboBio. The lentivirus-based shSURC-575, shSURC-1083, and shAPC (GenePharma) were used to infect SW620 and LoVo cell lines. The overexpression plasmid of SURC and APC were designed and purchased from GeneCopoeia. The miR–185-5p inhibitor and miR–185-5p mimic were purchased from RiboBio. Actinomycin D was purchased from MedChemExpress, while PNU-74654 and SKL2001 were purchased from Selleck.

### RNA extraction and quantification qPCR.

TRIzol reagent (Thermo Fisher Scientific) was used to obtain lysates of tissues and cell lines. Chloroform was then added and mixed and was centrifuged at 4°C for 15 minutes. Isopropanol and alcohol were added gradually and centrifuged. We then poured out the supernatant, dried the ethanol, and added the appropriate amount of RNase free water. The purity and concentration of RNA were detected by a nanodrop 2000 Ultradifferential photometer. A total of 1 μg of RNA was reverse-transcribed into cDNA by using the PrimeScript RT Reagent Kit according to the manufacturer’s protocol. Then, SYBR Green Master Mix was used to perform a qPCR reaction at 95°C for 600 seconds, followed by 42 cycles of 95°C for 5 seconds and 58°C for 30 seconds. The expression levels of GAPDH, β-actin, and U6 snRNA were used as normalizers in the in vivo and vitro experiments. The relative level of gene expression was calculated using the 2^−ΔΔCt^ formula. The sequences of primers used in the present study were supplied in Supplemental Table 1.

### Cellular proliferation and colony formation assay.

The cultured cells were digested by trypsin and plated in 96-well plates (2000 cells/well). For *cell counting kit-8 (*CCK8) analysis, the CCK8 reagent (DoJinDo) was diluted in the proportion of 1:10 and added to the cells cultured in 96-well plates at 37°C for 4 hours. Next, the absorbance was detected at a wavelength of 450 nm using a microplate spectrophotometer. For the colony formation experiment, the cultured cells were seeded into 6-well plates (2000 cells/well) for 7–10 days. The cells in the plates were then fixed with 4% paraformaldehyde and stained with crystal violet (Beyotime). The number of colonies was counted by ImageJ software (NIH).

### BrdU assays.

The cells were cultivated in 6-well plates (1 × 10^5^ cells/well) and incubated with BrdU (Merck) at 37°C for 30 minutes Then, cells were completely covered with cold 70% ethanol for 5 minutes, after which 1.5 M of HCl was added and incubated for 30 minutes at room temperature. After that, blocking buffer was added for 1 hour and primary Ab was incubated overnight at 4°C. The cells were incubated with fluorochrome-conjugated secondary Ab for 2 hours at room temperature in the dark. Finally, the cells were stained by 4,6-diamidino-2-phenylindole (DAPI, Beyotime) and counted under fluorescence microscopy.

### Flow cytometry for cell cycle.

A cell cycle detection kit (Beyotime) was used to carry out the cell cycle experiment according to the manufacturer’s instructions. First, the cells were collected in centrifugal tubes and fixed in 70% ethanol at 4°C for 24 hours. After that, the cells were incubated with propidium iodide and RNase for 30 minutes. Finally, a flow cytometer was used to analyze cell cycle distribution.

### Western blotting.

Protein lysates were extracted from the cells and tissues using RIPA lysis buffer (Beyotime) supplemented with Phosphatase Inhibitor Cocktail (Roche) and a phosphatase inhibitor (Roche). Then, a BCA assay kit (Thermo Fisher Scientific) was used to quantify the protein concentrations according to the manufacturer’s recommendations. After that, 10 μg of total protein lysates were separated by SDS-PAGE gel and transferred to PVDF membranes (Invitrogen) for 60 minutes at 100 V. Membranes were blocked in 5% nonfat milk powder for 2 hours and then incubated with anti-CCND2 (1:1000; Cell Signaling Technology, 3741S), anti-GAPDH (1:10000; ABclonal, AC002) or anti–β-catenin (1:10000; ABclonal, AC026) overnight at 4°C. After primary Ab incubation, the membranes were washed and incubated with anti-mouse (1:5000; ZSGB-BIP, ZB-2305) or anti-rabbit (1:5000; ZSGB-BIP, ZB-2301) HRP–conjugated secondary Abs for 1 hour. After the treatment of the ECL substrate (Thermo Fisher Scientific), the blotting images were captured by iBright CL1000 Instrument (Invitrogen).

### IHC.

Mouse and human tissues were embedded in paraffin and cut into 5 μm slices. Paraffin-embedded slices were dewaxed and rehydrated in xylene and alcohol. The slices were then boiled in 0.01 M of citrate buffer (pH 6.0) for 5 minutes. Next, 3% hydrogen peroxide was performed to eliminate endogenous peroxidase for 15 minutes, followed by a subsequent treatment with goat serum for 20 minutes at room temperature. Slices were incubated with anti-CCND2 (1:100; Cell Signaling Technology, 3741S) and anti-ki67 (1:100; Abcam, ab16667) Abs overnight at 4°C. After being treated with the secondary Ab and HRP, the DAB staining (MXB Biotechnologies) was used for IHC detection.

### Fluorescence in situ hybridization.

Cells were fixed with 4% paraformaldehyde at room temperature for 10 minutes and permeabilized with penetrant solution (PBS containing 0.5% Triton X-100) at 4°C for 5 minutes. After that, probes were added for hybridization at 37°C overnight. After DAPI staining, images were obtained with a fluorescence microscope. FISH probes and lncRNA FISH Kits were designed and purchased from RiboBio (Guangzhou).

Tissues were fixed overnight in 4% paraformaldehyde and cut into 5 μm slices. The tissue sections were immersed in xylene and dewaxed twice at room temperature for 20 minutes and placed in 100% ethanol, 85% ethanol, and 70% ethanol for 2 minutes. The sections were then boiled in H_2_O for 3 minutes. After that, the tissue sections were digested in pepsin solution (Merck) for 30 minutes, immersed in 0.2 M HCl solution for 10 minutes, and dehydrated in gradient ethanol. The probe was added for hybridization at 83°C for 5 minutes at 42°C overnight. The sections were washed, dyed with DAPI, and observed under the fluorescence microscope. The Olympus IX83 automatic microscopic optical platform was used for taking images and the ImageJ software was used to quantify RNA FISH images.

### ChIRP assay.

Beads used in this paper were purchased from Invitrogen and the ChIRP experiment was performed according to the protocol. Briefly, cells were collected and crosslinked with 1% glutaraldehyde solution to maintain the interaction of RNA-chromatin. The cells were lysed and the lysates were treated with ultrasonic crushing so that most DNA was interrupted into 100–500 bp in length. The probe purchased from RiboBio was incubated with the cell lysate at 37°C for 4 hours, after which magnetic beads were added to separate the probe. Finally, RNA fragments were extracted, purified, and quantified by qPCR.

### ChIP.

A SimpleChIP Plus Enzymatic Chromatin IP Kit (Cell Signaling Technology) was used to carry out the experiment according to the manufacturer’s instructions. We first added formaldehyde to the cell culture dishes to crosslink the protein with DNA. After that, glycine was added to terminate the crosslinking and the cells were scraped off the plate. Then, the DNA was digested to a length of about 150–900 bp, and the nucleus was completely cleaved using an ultrasonic crusher. Abs were added to the lysate at 4°C overnight. The Abs used in this paper include Normal Rabbit IgG (Cell Signaling Technology, 2729), Histone H3 Rabbit mAb (Cell Signaling Technology, 4620), and anti–β-catenin (Abcam, ab32572). ChIP-Grade Protein G Magnetic Beads (Cell Signaling Technology, 9006) were added to the sample and incubated at 4°C for 2 hours. The chromatin was eluted by gently swirling, mixing, and incubating at 65°C for 30 minutes, followed by adding NaCl and proteinase K at 65°C for 2 hours. Finally, DNA was purified and detected by qPCR.

### Luciferase reporter assays.

For luciferase reporter experiments, 293T cells were digested and seeded into 6-well plates (5 × 10^5^ cells/well). Luciferase reporter constructs were cotransfected into 293T cells with related expression plasmid using Lipo-fectamine 3000 Reagent (Thermo Fisher Scientific). After 48 hours, the cells were lysed by adding the appropriate amount of reporter gene lysate. Then, Firefly luciferase and Renilla luciferase were added to detect the luciferase activities. The Dual Luciferase Reporter Assay kit (GeneCopoeia) was used to detect the luciferase activity according to the manufacturer’s instructions.

### TCGA and RNA-Seq analysis.

Gene expression data of normal colorectal tissues and colorectal tumor samples were downloaded from the Genomic Data Commons (GDC, https://portal.gdc.cancer.gov). RNA-Seq was performed by Illumina HiSeq 2000 to screen out differentially expressed genes. Differentially expressed genes were defined when the fold change was greater or equal to 1 and the adjusted *P* value was less than 0.05 between different groups. The data of RNA-Seq has been submitted to the China National Center for Bioinformation (CNCB, https://www.cncb.ac.cn/), and the accession number of the submission is HRA002385.

### Statistics.

GraphPad Prism version 7.0 was used for mapping and statistical analysis in this study. An unpaired or paired Student’s *t* test, ANOVA, or Log-Rank test (Kaplan-Meier curves) were used to determine statistical significance. The sample size and the number of experimental repetitions were noted in the figure legends.

### Study approval.

Our experiments were approved by the West China Hospital of Sichuan University Biomedical Research Ethics Committee (year 2018 [serial number 280]) and performed in compliance with Sichuan University guidelines concerning animal use and care. All patients gave written informed consent to participate in the study.

## Author contributions

JL, YJ, and NC were involved in acquisition of the data with assistance from HW, ZD, JF, DZ, WZ, LH, RJ, and XS. HD and LD developed the study concept and design and obtained funding. CF, XY, and ZJ were involved in human colon cancer tissue collection. CF conducted the prognosis analysis. LC and GS analyzed and interpreted the data. HZ conducted the statistical analysis. JL, LD, and HD drafted the manuscript and conducted a critical revision of the manuscript for important intellectual content.

## Supplementary Material

Supplemental data

## Version 1 05/26/2022

In-Press Preview

## Version 2 08/08/2022

Electronic publication

## Version 3 09/29/2022

Corrected publication date in PDF version
