# Supplementary material for: A specific upregulated long noncoding RNA in colorectal cancer promotes cancer progression
Source: JCI Insight. 2022 Aug 8;7(15):e158855. doi: 10.1172/jci.insight.158855 (PMC9462503; doi:10.1172/jci.insight.158855)
Supplement: Supplemental data [file jciinsight-7-158855-s009.pdf]

Supplementary Figures and Figure Legends

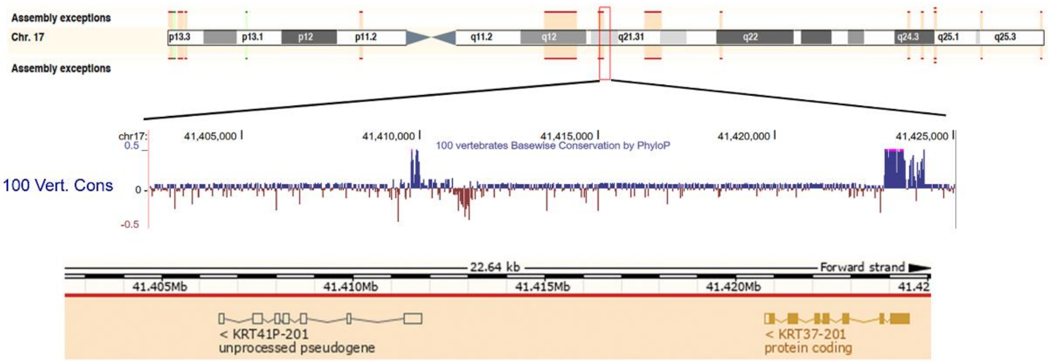

Supplementary Figure 1 The location of lncRNA-SURC.

The location of SURC in chromosome and its conservation analysis among species.

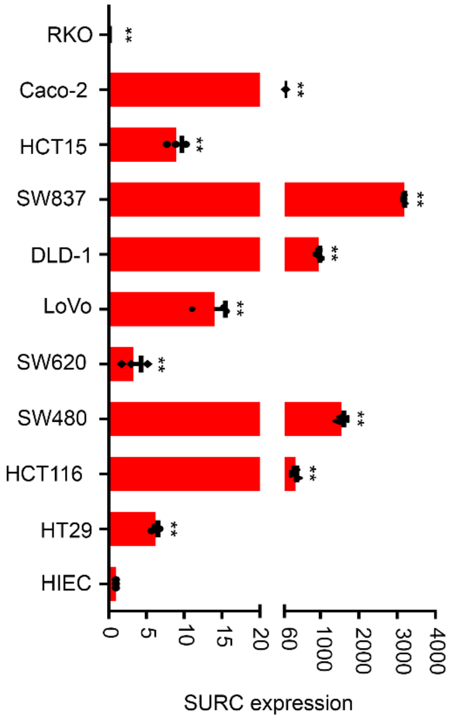

Supplementary Figure 2 Expression of SURC in CRC cells.

qPCR detected the expression of SURC in CRC cells and normal intestinal epithelial cell (n=3, **, $P < 0.01$). All results represent the mean ± SEM. Statistical differences were calculated by using one-way ANOVA and Dunnett's multiple-comparison test.

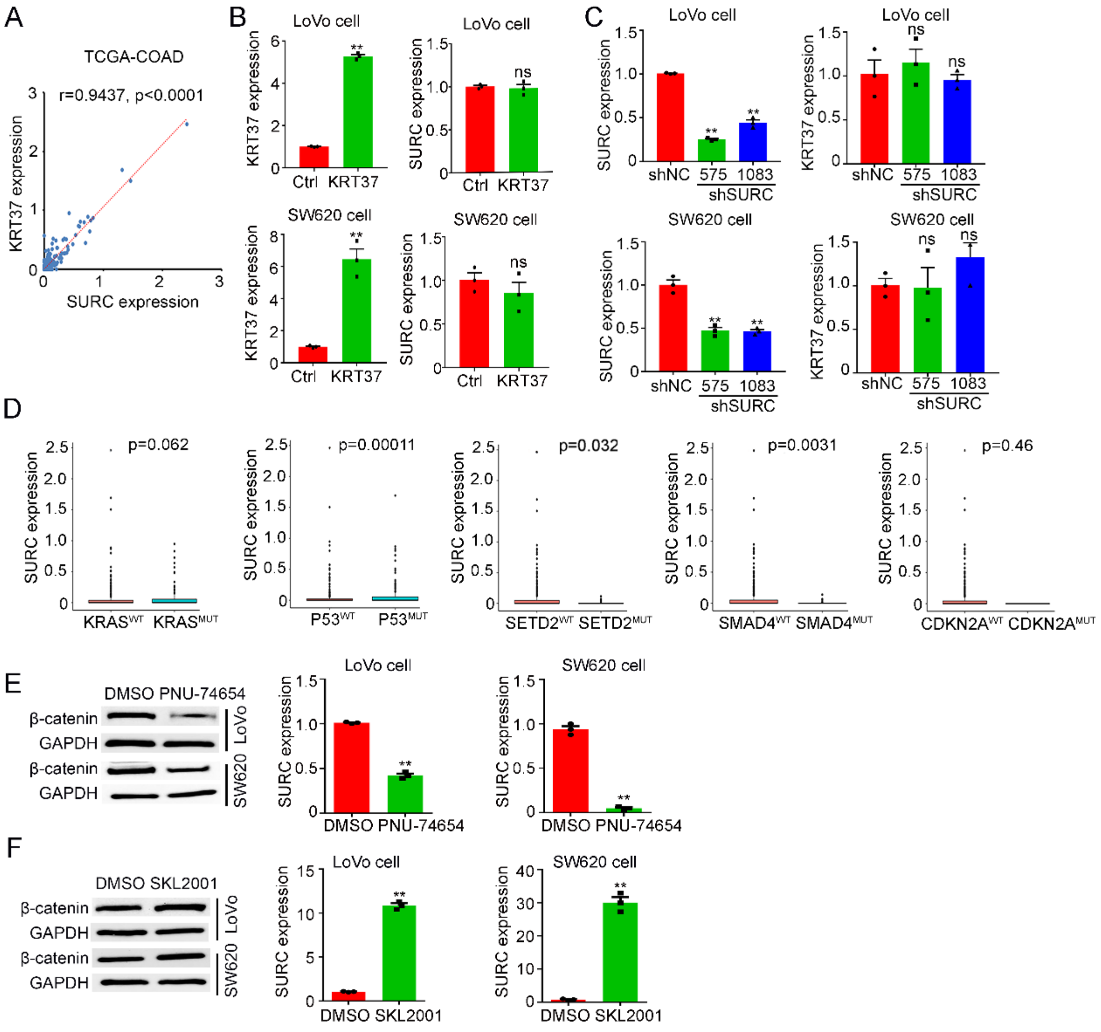

Supplementary Figure 3 KRT37 did not affect SURC expression.

(A) KRT37 expression was positively correlated with SURC expression in TCGA-COAD database. The r values and P values are from Pearson's correlation analysis. (B) Over-expression of KRT37 in SW620 and LoVo cells did not affect the expression of SURC by qPCR (n=3, **, P < 0.01). (C) SW620 and LoVo cells were infected with lenti-shNC or lenti-shSURC and detected the expression of KRT37 by qPCR (n=3, **, P < 0.01). (D) The analysis of SURC expression in the mutation of KRAS, P53, SETD2, SMAD4 and CDKN2A compared with WT in TCGA database. (E) Western blotting shows the expression of β-catenin in SW620 and LoVo cells treated with PNU-74654 and DMSO. GAPDH was used as a loading control. qPCR shows the expression of SURC in SW620 and LoVo cells treated with PNU-74654 and DMSO (n=3, **, P <

0.01). (F) Western blotting shows the expression of β-catenin in SW620 and LoVo cells treated with SKL2001 and DMSO. GAPDH was used as a loading control. qPCR shows the expression of SURC in SW620 and LoVo cells treated with SKL2001 and DMSO (n=3, **, P < 0.01). All results represent the mean ± SEM. Statistical differences were calculated by using one-way ANOVA and Dunnett's multiple-comparison test (for C) and unpaired Student's *t* test (for B and D-F).

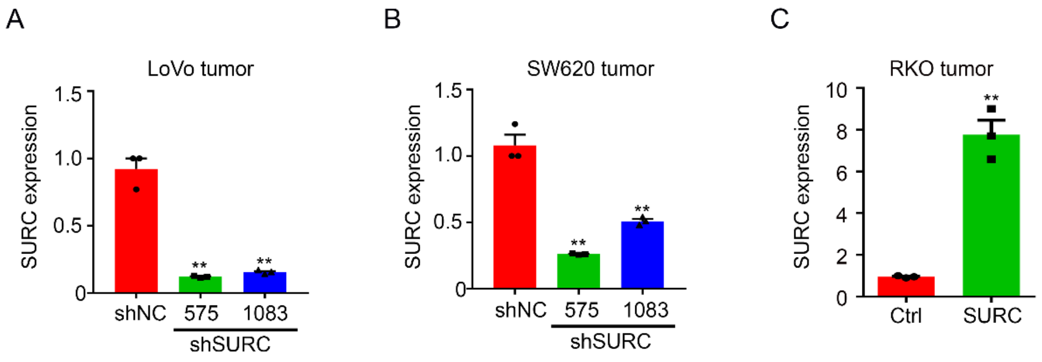

Supplementary Figure 4 The SURC expression was detected in knockdown and overexpression tumors.

(A&B) qPCR determined the expression of SURC in subcutaneous tumors of LoVo and SW620 cells (n=3, **, P < 0.01). (C) The expression of SURC was detected in subcutaneous tumors of RKO cells (n=3, **, P < 0.01). All results represent the mean ± SEM. Single comparisons to Ctrl were made using one-way ANOVA and Dunnett's multiple-comparison test (for A and B) and unpaired Student's *t* test (for C). The level of Ctrl and shNC were arbitrarily set to 1.

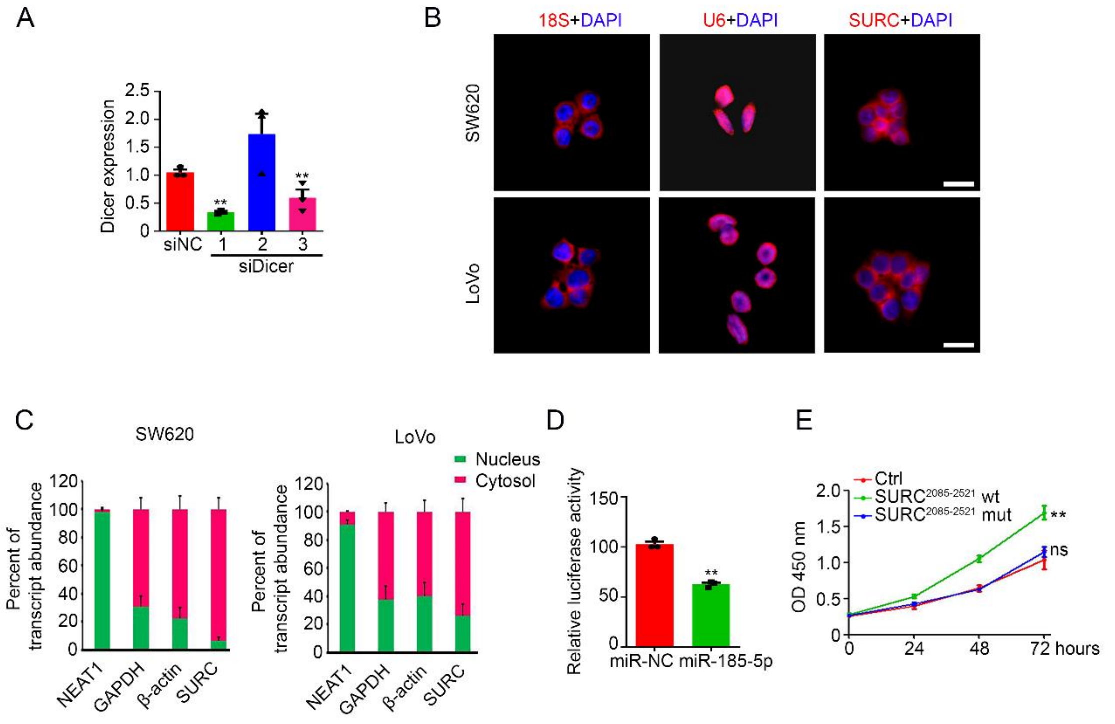

Supplementary Figure 5 Direct binding of SURC and miR-185-5p.

(A) The knockdown efficiency of siDicer was detected by qPCR (n=3, **, P < 0.01). (B) FISH assays identify the subcellular localization of SURC (red) in SW620 and LoVo cells, blue represents DAPI, Scale bar represents 20 μm. (C) Subcellular localization of SURC in the cytoplasmic and nuclear extractions from SW620 and LoVo cells by qPCR. (D) Dual-luciferase reporter system experiment confirmed the combination of CCND2 and miR-185-5p (n=3, **, P < 0.01). (E) CCK8 assay shows the function of SURC core region in RKO cells which was transfected with $SURC^{2085-2521}$WT or MUT plasmid (n=5, **, P < 0.01; ns, no significant difference). All results represent the mean ± SEM. Statistical differences were calculated by using one-way ANOVA and Dunnett's multiple-comparison test (for A and E) and unpaired Student's *t* test (for D).

.

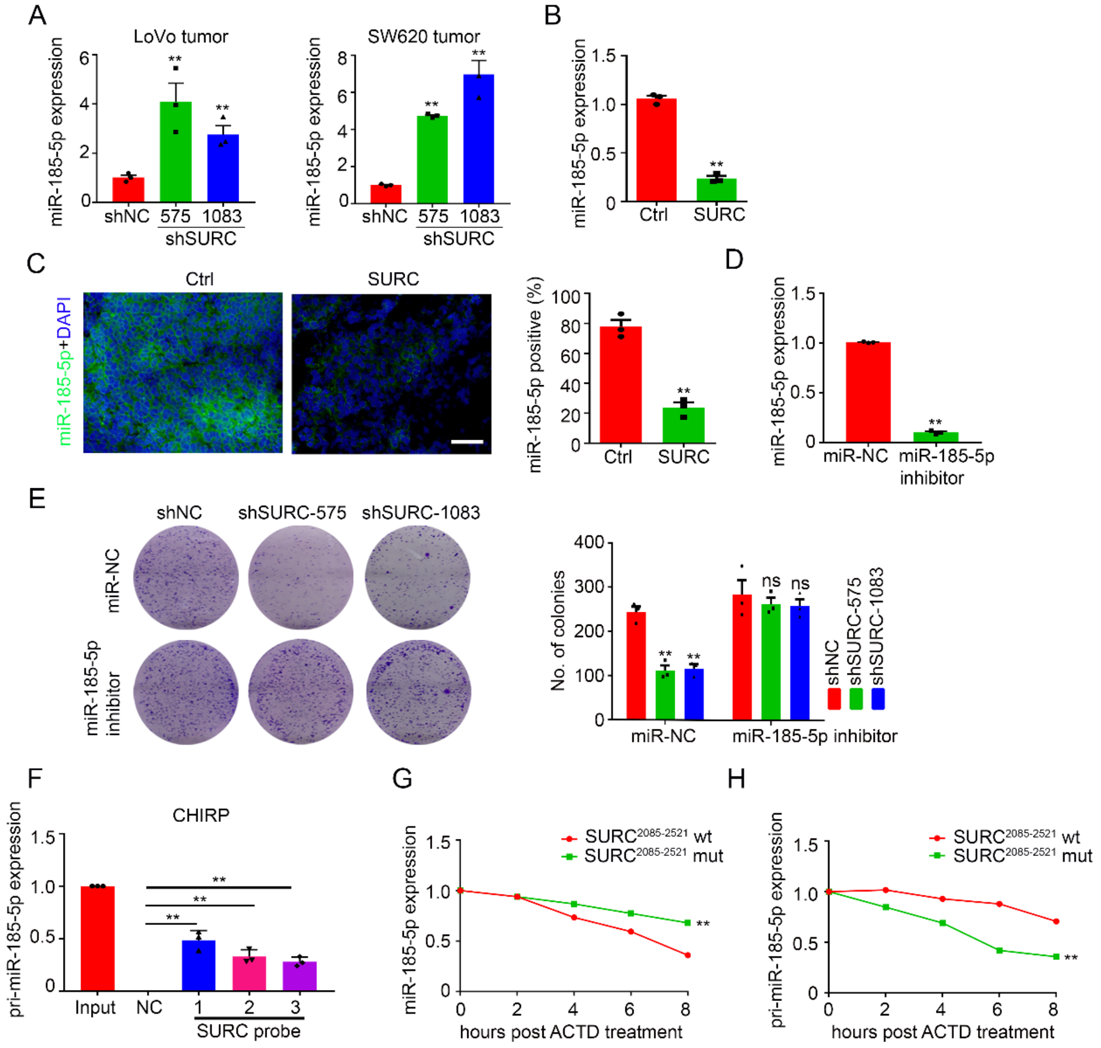

Supplementary Figure 6 The interaction of SURC and miR-185-5p affected cell growth.

(A) miR-185-5p expression in subcutaneous tumors of SW620 and LoVo by qPCR (n=3, **, P < 0.01). (B) miR-185-5p expression in subcutaneous tumors of RKO cells (n=3, **, P < 0.01). (C) The expression of miR-185-5p was detected in subcutaneous tumors by FISH (Scale bar represents 100 μm). Analysis of miR-185-5p positive cells in each frame (n=3, **, P < 0.01). (D) qPCR shows the expression of miR-185-5p after administration with miR-185-5p inhibitor (n=3, **, P < 0.01). (E) Colony formation was examined in LoVo shSURC and shNC cells after administration with miR-185-5p inhibitor (n=3, **, P < 0.01; ns, no significant difference). (F) ChIRP assay detected the

ability of SURC direct binding to pri-miR-185-5p in SW620 cells (n=3, **, P < 0.01). (G) Levels of miR-185-5p and (H) pri-miR-185-5p were examined at different times after administration with actinomycin d in SURC knockdown SW620 cells transfected with SURC$^{2085-2521}$ WT or MUT plasmid (n=3, **, P < 0.01). All results represent the mean ± SEM. Statistical differences were calculated by using one-way ANOVA and Dunnett's multiple-comparison test (for A, E and F) and unpaired Student's *t* test (for B-D, G and H).

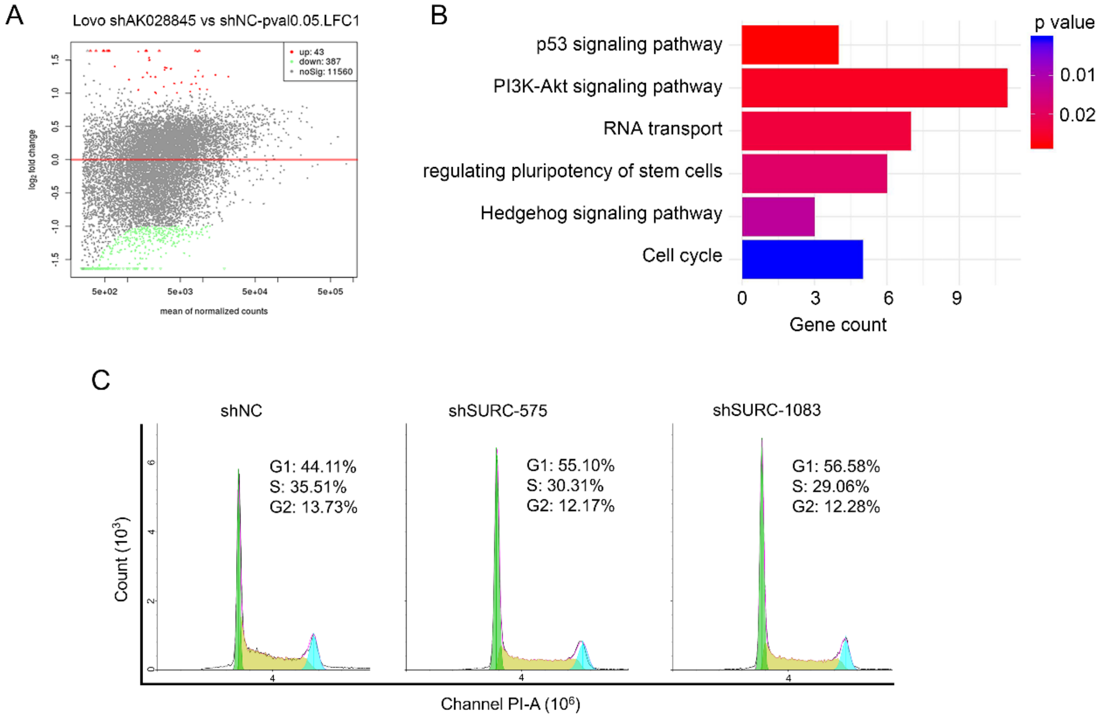

Supplementary Figure 7 Regulation of downstream genes by SURC.

(A) Volcano plot shows significantly deregulated lncRNAs in LoVo shSURC cells compared with shNC cells based on RNA sequencing. (B) Pathway analysis of deregulated genes in LoVo shSURC-treated cells (compared with LoVo WT cells). (C) The cell cycle was analyzed by flow cytometry analysis in LoVo cells.

Supplementary Table 1 Sequences of the primer used in the present study

Primer names	Sequences
SURC Homo For	5'-TAAACCACTGGAGAGCGGATA-3'
SURC Homo Rev	5'-TTCTGTCTCATGTGGCATCG-3'
APC Homo For	5'-AAGCATGAAACCGGCTCACAT-3'
APC Homo Rev	5'-CATTCGTGTAGTTGAACCCTGA-3'
SURC-pro1 Homo For	5'-CGAGGGGCTAACCTGGAAAAT-3'
SURC-pro1 Homo Rev	5'-TTACGCCTTGCAGGACACTC-3'
SURC-pro2 Homo For	5'-GGCCAAGATGGGTCCTCAAT-3'
SURC-pro2 Homo Rev	5'-AAATGCCCATCTTACACCTTCT-3'
SURC-pro3 Homo For	5'-TCCCCTACCCACTCCATTTCT-3'
SURC-pro3 Homo Rev	5'-ACCTGAAATGGCTTCATCCCC-3'
Dicer Homo For	5'-TCGCTTGCTGGTGCCATTTA-3'
Dicer Homo Rev	5'-AGTGACTCTGACCTTCCCGT-3'
CCND2 Homo For	5'-AGCTGTGCATTTACACCGAC-3'
CCND2 Homo Rev	5'-CATGCTTGCGGATCAGAGAC-3'
KRT37 Homo For	5'-CAGAGAACCGCTACTGCGT-3'
KRT37 Homo Rev	5'-GTGGTCACCGGTTCTTTCTTG-3'
NEAT1 Homo For	5'-CCACAACGCAGATTGATGCC-3'
NEAT1 Homo Rev	5'-GAAACGCACAAGAAGGCAGG-3'
ACTB Homo For	5'-TGCGTGACATTAAGGAGAA-3'
ACTB Homo Rev	5'-AAGGAAGGCTGGAAGAGT-3'
GAPDH Homo For	5'-TCAAGGCTGAGAACGGGAAG-3'
GAPDH Homo Rev	5'-TCGCCCCACTTGATTTTGGA-3'
AK028845 Mus For	5'-GTCATCAGCAGCCAGCTTGG-3'
AK028845 Mus Rev	5'-AGAGGCATCTGTGAGCTGGCTT-3'
ACTB Mus For	5'-ATCGCTGCGCTGGTCG-3'
ACTB Mus Rev	5'-CCACGATGGAGGGGAATACAG-3'
